# Stochastic Propagation of Fatigue Cracks in Welded Joints of Steel Bridge Decks under Simulated Traffic Loading

**DOI:** 10.3390/s23115067

**Published:** 2023-05-25

**Authors:** Naiwei Lu, Jing Liu, Honghao Wang, Heping Yuan, Yuan Luo

**Affiliations:** 1 School of Civil Engineering, Changsha University of Science and Technology, Changsha 410114, China; lunaiwei@csust.edu.cn (N.L.);; 2 Hunan Province Research Center for Safety Control Technology and Equipment of Bridge Engineering, Changsha University of Science and Technology, Changsha 410114, China; 3 College of Civil Engineering, Hunan University of Technology, Zhuzhou 412007, China

**Keywords:** orthotropic steel decks, fatigue crack, stochastic propagation, stochastic traffic flow, linear elastic fracture mechanics

## Abstract

The fatigue cracking of orthotropic steel bridge decks (OSDs) is a difficult problem that hinders the development of steel structures. The most important reasons for the occurrence of fatigue cracking are steadily growing traffic loads and unavoidable truck overloading. Stochastic traffic loading leads to the random propagation behavior of fatigue cracks, which increases the difficulty of the fatigue life evaluations of OSDs. This study developed a computational framework for the fatigue crack propagation of OSDs under stochastic traffic loads based on traffic data and finite element methods. Stochastic traffic load models were established based on site-specific, weigh-in-motion measurements to simulate fatigue stress spectra of welded joints. The influence of the transverse loading positions of the wheel tracks on the stress intensity factor of the crack tip was investigated. The random propagation paths of the crack under stochastic traffic loads were evaluated. Both ascending and descending load spectra were considered in the traffic loading pattern. The numerical results indicated that the maximum value of K_I_ was 568.18 (MPa·mm^1/2^) under the most critical transversal condition of the wheel load. However, the maximum value decreased by 66.4% under the condition of transversal moving by 450 mm. In addition, the propagation angle of the crack tip increased from 0.24° to 0.34°—an increase ratio of 42%. Under the three stochastic load spectra and the simulated wheel loading distributions, the crack propagation range was almost limited to within 10 mm. The migration effect was the most obvious under the descending load spectrum. The research results of this study can provide theoretical and technical support for the fatigue and fatigue reliability evaluation of existing steel bridge decks.

## 1. Introduction

Steel structures have the following advantages: they are very strong, convenient to construct, lightweight, and have good mechanical properties, which make them the primary structural forms in large infrastructures. The orthotropic steel deck (OSD) is the main type of deck for long-span steel bridges, especially for cable-supported bridges. However, fatigue cracking is a key problem for the service safety and the serviceability of OSD structures. This is a result of low local stiffness, welding defects and heavy traffic loads [1,2]. Fatigue cracking is a problem in many famous long-span bridges, such as the Humen Bridge, the Jiangyin Bridge, and the Junshan Bridge [3,4]. Of all the various types of fatigue cracks, the most critical and common cracking type is in the welding roots of the rib-to-deck connections. In addition, the cracks in welding roots are usually under the pavement, making them difficult to detect [5]. The fatigue performance of welded joints is affected by multiple uncertainties, such as base metal defects, penetration rate, residual stresses, and the random distribution of traffic load. These uncertainties result in a high degree of randomness in fatigue cracking. Because of these uncertain factors, the crack propagation path randomly changes [6,7]. However, the traditional deterministic crack growth analysis method is cannot to describe the random crack propagation characteristics effectively. In addition, the research results of this study into the random propagation behavior of fatigue cracks on steel bridge decks could lay a theoretical foundation for their fatigue reliability evaluations and optimal designs.

Many theoretical and experimental studies have been carried out to investigate the defect initiation, crack propagation, and fatigue fractures in rib-to-deck welded joints. In the numerical simulation of fatigue cracks, typical two-dimensional, semi-elliptic, type-I crack, with fixed proportions for the long and short axes, were widely utilized to study crack propagation behavior. Wang et al. [8] observed the three-dimensional propagation behavior of fatigue cracks in steel bridge decks due to the high strain energy of the type-II and type-III characteristics in the later period. Di et al. [9] developed a fatigue evaluation approach for in-service steel bridge decks, based on the strain monitoring data. Wang et al. [10] evaluated the distortion-induced fatigue crack growth rate using the extended finite element method.

Since the mechanical behavior of fatigue cracks in OSDs is determined by three different crack types, the maximum circumferential stress and minimum strain energy density are commonly utilized to judge the spatial crack propagation behavior. Zhang et al. [11] proposed a simulation method for three-dimensional, semi-elliptic crack propagation at rib-to-deck welded joints, which was verified by an experimental test. Rodenburg et al. [12] developed an approach for evaluating the growth rate of both penetrating and non-penetrating cracks. Mahmood et al. [13] investigated the propagation behavior of penetrating fatigue cracks in the I-beams of a steel bridge, based on an advanced equivalent stress intensity approach. Cheng et al. [14] investigated the crack propagation behavior of rib-to-floor, beam-welded connections in ultra-high performance, concrete-reinforced OSDs, which were subjected to longitudinal flexural loads. Huang et al. [15] investigated the propagation characteristics and fatigue life of rib-to-diaphragm welded joints under a constant amplitude load. Fang et al. [16] investigated the fatigue failure mechanism and optimization of double-sided welds in OSDs. Cui et al. [17] developed fatigue mechanics for a new compression-compression zone for welded joints in OSDs. Xu et al. [18] investigated a three-dimensional weight function method for the rapid calculation of crack stress intensity factors.

In this study, we developed a computational framework for predicting fatigue crack propagation in the welded joints of OSDs under stochastic traffic loading. Stochastic traffic load models were established based on site-specific traffic data, for the purpose of simulating the fatigue stress spectra of welded joints. The influence of the transverse loading position of trucks’ wheels on the stress intensity factor of the crack tip was also investigated. The random propagation paths of the crack under stochastic traffic loading were evaluated. Both ascending and descending load spectra were considered in the traffic loading pattern. The research results can provide a theoretical basis for the fatigue reliability of existing steel bridge decks, based on practical traffic loading.

## 2. Theoretical Basis of Fatigue Crack Propagation

### 2.1. Theoretical Basis of Linear Elastic Fracture Mechanics

Linear elastic fracture mechanics (LEFM) utilize a stress intensity factor, *K*, which is determined by a structural stress analysis to express the relationship between external loads and crack sizes. The LEFM has been widely used for the fatigue analysis of linear elastic materials and structures, including welded joints in ODSs [19,20,21]. It is well known that the fatigue crack growth rate in structures under cyclic loading is dominated by the stress intensity factor of the crack tip. The Paris formula describes the relationship between the crack growth rate and the stress intensity factor at the crack tip [22], which is written as:

(1) $$da/dN=C(\Delta K{)}^{n}$$

where *a* is the crack size; *N* is the number of cycle loads; *C* and *n* are material constants; and Δ*K* is the stress intensity range of the crack tip. Note that the fatigue crack stress intensity factor in engineering structures is usually variable because of the crack shape variation. For of the fatigue crack shape, the fatigue crack growth life, *N_f_*, can be calculated by the superposition of limited slight increments, which is written as:

(2) $$N_{f}=\sum_{i=1}^{r}\Delta N_{i}=\sum_{i=1}^{r}\left(\frac{\Delta a_{i}}{C{\left(\Delta K_{}\right)}_{i}^{n}}\right)$$

where the crack life is divided into *r* steps, and *i* = 1, 2, …, *r*; Δ*a_i_* is the crack increment of the *i*th step; Δ*K_i_* is the stress intensity range at the *i*th step; and Δ*N* is the number of load cycles with respect to the crack growth Δ*a*.

The Paris formula has been successfully applied to the residual fatigue life, the fatigue performance evaluation, and the maintenance and reinforcement of steel bridge decks during service. In addition, in accordance with the finite element and the fracture mechanics methods, many researchers have adopted numerical simulation methods to determine the dominant cracking modes and the propagation behaviors that control the macroscopic crack propagation life at typical structural details of steel bridge decks. It plays a key role in the research into the fatigue damage mechanisms of OSDs.

According to the Paris formula, the stress intensity factor of the crack tip is the key index in evaluating the crack propagation characteristics. Yau et al. [23] proposed the M-integral method for calculating the stress intensity factors with three fracture modes which were determined by three types of stress intensity factors, including *K_I_*, *K_II_*, and *K_III_*. The M-integral method is generally written as follow:

(3) $$M^{\left(1,2\right)}=\int_{\Gamma }(\sigma_{ij}^{\left(1\right)}\frac{\partial u_{i}^{\left(2\right)}}{\partial x_{1}}+\sigma_{ij}^{\left(2\right)}\frac{\partial u_{i}^{\left(1\right)}}{\partial x_{1}}-W^{\left(1,2\right)}\delta_{1j})\frac{\partial q}{\partial x_{j}}ds/A_{q}$$

where $A_{q}={\int {}_{L}q}_{t}ds$; *q*_t_ is the function value of the crack front; and *W*^(1,2)^ is the interaction strain energy density, which is defined as follow:

(4) $$W^{\left(1,2\right)}=\sigma_{ij}^{\left(1\right)}\varepsilon_{ij}^{\left(2\right)}=\sigma_{ij}^{\left(2\right)}\varepsilon_{ij}^{\left(1\right)}$$

where *σ_ij_* is the stress tensor; *ε_ij_* is the strain tensor; superscripts 1 and 2 represent the actual and auxiliary fields, respectively.

Based on Equations (3) and (4), the relationship among the M-integral, the material properties, and the stress intensity factor, *K,* can be written as follows:

(5) $$M^{\left(1,2\right)}=2\times \left[\frac{1-\nu^{2}}{E}K_{I}^{\left(1\right)}K_{I}^{\left(2\right)}+\frac{1-\nu^{2}}{E}K_{II}^{\left(1\right)}K_{II}^{\left(2\right)}+\frac{1+\nu^{2}}{E}K_{III}^{\left(1\right)}K_{III}^{\left(2\right)}\right]$$

where *E* is the elastic modules and *v* is the material Poisson parameter. Therefore, a comprehensive expression can be written as follows:

(6) $$\int_{\Gamma }(\sigma_{ij}^{\left(1\right)}\frac{\partial u_{i}^{\left(2\right)}}{\partial x_{1}}+\sigma_{ij}^{\left(2\right)}\frac{\partial u_{i}^{\left(1\right)}}{\partial x_{1}}-W^{\left(1,2\right)}\delta_{1j})\frac{\partial q}{\partial x_{j}}ds/A_{q}=2\times \left[\begin{array}{l} \frac{1-\nu^{2}}{E}K_{I}^{\left(1\right)}K_{I}^{\left(2\right)}+\frac{1-\nu^{2}}{E}K_{II}^{\left(1\right)}K_{II}^{\left(2\right)}\\ +\frac{1+\nu^{2}}{E}K_{III}^{\left(1\right)}K_{III}^{\left(2\right)} \end{array}\right]$$

where *K_I_*, *K_II_*, and *K_III_* can be calculated based on the finite element methods.

### 2.2. Crack Propagation Simulation Method Based on FRANC3D-ABAQUS Interactive Technology

As previously mentioned, the stress intensity factor, *K*, is usually estimated using finite element methods. Popular finite element software, such as ANSYS, ABAQUS, and NASTRAN, can be utilized to simulate the mechanical characteristics of the fatigue cracks in a structure. Complicated structural modeling mostly depends on finite element simulations, while fatigue cracking analysis usually depends on professional software. A commercial program, namely Fracture Analysis Code 3D (FRANC3D) can be used to compute the stress intensity factor for a three-dimensional crack propagation process. FRANC3D is widely used in aerospace engineering, mechanical engineering, and engineering structures [24,25]. The accuracy of its computational results is generally accepted by industry requirements.

**Figure 1:**
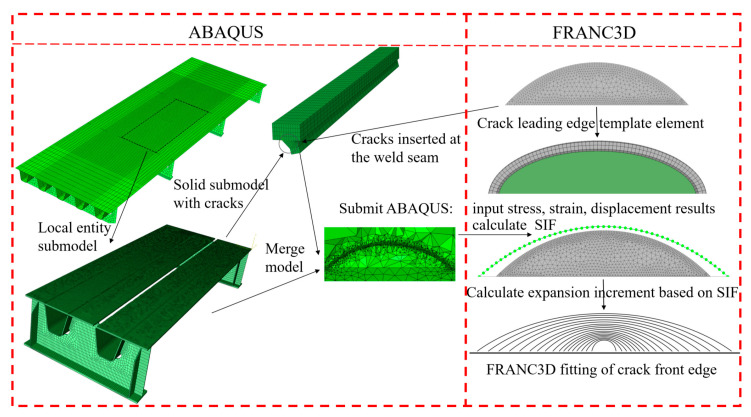
Computational framework for FRANC3D-ABAQUS.

Fatigue cracks should be inserted into the finite element model before the fracture mechanics analysis. Based on adaptive mesh repartitioning, initial cracks of any shape can be easily and efficiently introduced by FRANC3D. In addition, the efficiency and accuracy of complex structural computations can be significantly improved by combining them with the ABAQUS program. The principal steps for FRANC3D-ABAQUS interactive technology are the ABAQUS modeling analysis and the FRANC3D fracture mechanics analysis. The computational flowchart is shown in Figure 1.

The structural model established by ABAQUS contains different material attributes, mesh types and contact types. Therefore, the node sets and cell sets should be predefined in ABAQUS and subsequently inserted into FRANC3D. In other words, ABAQUS provides a detailed, global structural model, and FRANC3D provides an enhanced, local crack model. However, it is necessary to ensure that the sub-model is not affected by the stress concentration area. The details of these procedures are described below.

**Figure 2:**
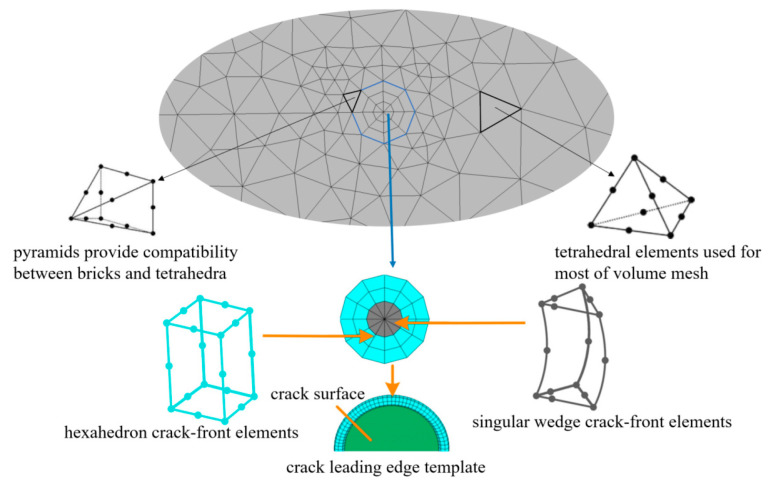
Crack-leading edge-template element.

1. Establish the structural, global finite element model: To achieve the objective of our current investigation, a segmental model of the steel bridge deck was built in ABAQUS. Subsequently, a network partition and a boundary condition were conducted.
2. Establish a detailed, local sub-model: Subdivide the overall model based on FRANC3D and define the unit’s set group in the crack propagation area as a sub-model. Subsequently, generate the solid, local sub-model of the steel bridge deck, and re-mesh the solid, local sub-model. The re-meshed sub-model will be connected with the global model and the finite element analysis can then be conducted.
3. Insert the initial cracks: Initially, define the crack parameters, such as the crack positions, shapes, and directions. Next, introduce the initial fatigue crack into the structural details of the steel bridge deck’s roof-U rib weld using the crack propagation professional analysis software, FRANC3D. This establishes a solid, local sub-model, which contains the initial crack defect. In this study, after the cracks were introduced, the FRANC3D software automatically re-meshed the local sub-model, and established a refined local sub-model of the entity with cracks. The updated sub-model grid size was 0.025 mm. After this stage, the mesh of the crack tip is then re-divided and adjusted to generate a regular, three-turn unit ring. Among them, the innermost one is a 1/4 node, wedge-shaped unit and the outer two circles are second-order hexahedral unit rings.
4. Conduct the finite element computation: Insert the solid sub-model, which contains the initial crack defect, and the remaining parent model into the global model. The FRANC3D software will then perform the integral conservation calculations on the two element rings in the integral domain around the crack tip, which are an inner-ring, singular wedge element and an outer-ring, hexahedron element. Eventually, the following results will be provided: the crack tip stress field, the strain field, and the displacement results.
5. Conduct the stress intensity factor computation: Use the M-integral method to calculate the stress intensity factor of the crack tip. FRANC3D will arrange three-unit rings at the crack tip. The conservation integration will be conducted around the first- and second- unit rings around the crack tip. In the calculation, the unit ring of the third circle is not included in the integral domain; therefore, it does not participate in the calculation. The arrangement of the unit ring at the crack tip is shown in Figure 2.

In Figure 2, the volume mesh in the crack front template is pre-defined. When the volume mesh is divided, a regular, three-circle unit ring will be generated around the crack front, inside the template. The grid of the crack tip will be re-divided and adjusted, and a quarter-node, singular unit is used to represent the crack tip node to adapt to the singularity of the stress field and the displacement field at the crack tip. The crack front is divided into three-layer grids, including a combination of the inner-ring, a singular wedge unit and the outer, two-ring hexahedral units. The inner ring of the crack front layer is a quarter, 15-node singular wedge unit, and the outer two-ring is a quarter, 20-node, hexahedron unit-ring unit.

The accuracy and feasibility of the FRANC3D-ABAQUS interactive technology was verified in this study by computing the stress intensity factor, *K,* of the fatigue crack. Wang et al. [26] calculated the stress intensity factors of the cracks in steel wires and these were validated by experimental results. Many researchers [27] have indicated that FRANC3D-ABAQUS interactive technology is feasible in calculating the stress intensity factor of three-dimensional fatigue cracks, with a maximum error of less than 2.0%.

## 3. Stochastic Traffic Load Simulation Based on Site-Specific Traffic Data

### 3.1. Description of the WIM System and Traffic Data

**Figure 3:**
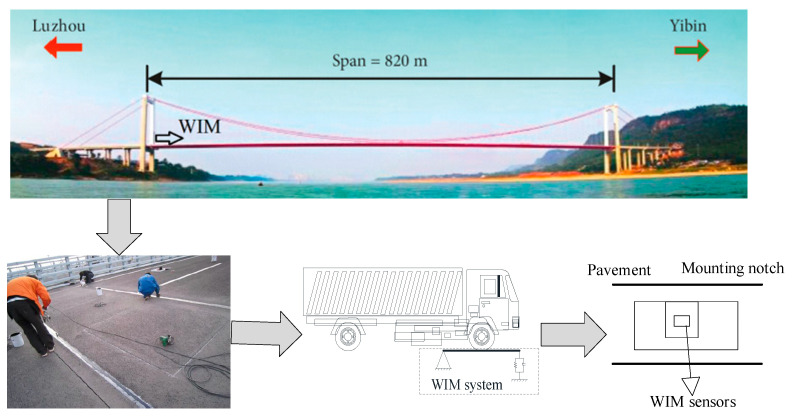
Description of the WIM system of Nanxi Yangtze River Bridge.

The prototype bridge used in this study was the Nanxi Yangtze River Bridge in Sichuan, China. The bridge was installed using standard structural health monitoring systems, including a traffic weigh-in-motion (WIM) system [28,29]. A statistical analysis of the traffic flow parameters of the Nanxi Yangtze River Bridge was conducted based on the traffic data. A description of the WIM system is shown in Figure 3. It is observed that the WIM system works in the same way as a beam that captures the axle weight of each passing vehicle, dynamically. There are four weighting platforms, in accordance with the four driving lanes. In addition to the vehicle weight and the driving lane, the axle spacing, and the passing time can also be recorded, from which the vehicle configuration and the driving speed can be deduced.

Table 1 shows a sample of the traffic data that was taken from the WIM system. More traffic data can be found from the journal website. These data confirmed that many extremely overloaded trucks were captured by the system. The largest recorded gross vehicle weight (GVW) was 165 t, which is three times over the legal GVW in China. Table 2 summarizes the details of the overloaded trucks. In the present study, these traffic data were utilized to estimate probabilistic models of the traffic parameters. Subsequently, stochastic traffic load models were simulated to evaluate the fatigue crack propagation behavior and fatigue life of steel bridge decks.

### 3.2. Probabilistic Modeling of Traffic Parameters

There are many types of vehicle parameters on a highway, such as driving speeds and gross weights. It is necessary to consider the classification of vehicles comprehensively, according to the characteristics of the traffic flow and normative standards. The vehicle types generally follow a uniform distribution. The traffic data selected for this study were selected over a 31-day period, between 1 July and 31 July 2015. The total number of vehicles was 241,968 and the average daily traffic was 7805. The vehicle type share of the vehicles in every lane was analyzed. The proportions of the vehicle types in the different driving lanes are shown in Table 3.

The vehicle spacing is another important parameter that impacts the density of traffic loading. The distance between the vehicles is random due to the influence of time and vehicle speed. Although there is no intuitive distance information data, the distance can be calculated using its speed and time interval in the WIM system. The specific equation is expressed as follows:

(7) $$S_{i}=V_{i}*(T_{i+1}-T_{i})$$

where *V_i_* represents the speed of the *i*th vehicle passing the measurement point; *T_i_* represents the time of the *i*th vehicle passing the measurement point; and *T_i_*_+1_ represents the time of the *i*th + 1 vehicle passing the measurement point.

**Figure 4:**
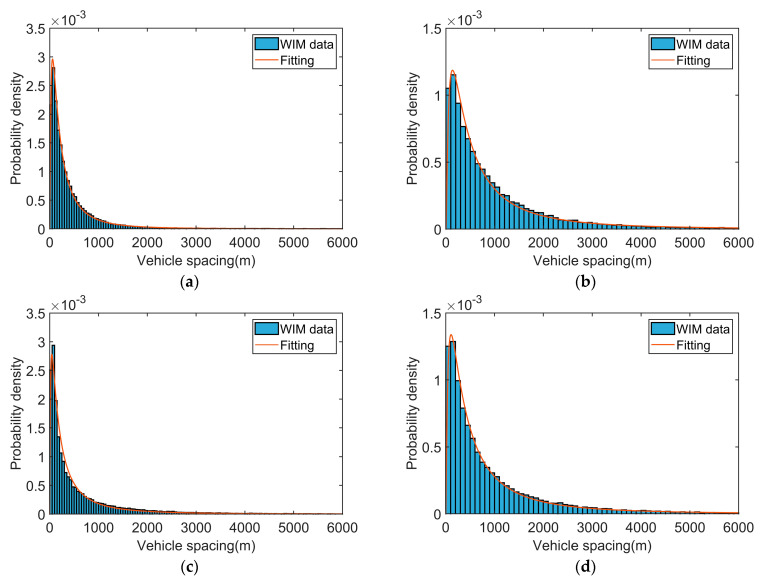
Probability distribution of vehicle distance in different lanes: (**a**) Lane 1. (**b**) Lane 2. (**c**) Lane 3. (**d**) Lane 4.

The vehicle spacing in each driving lane was calculated using Equation (6) and was fitted to the probability functions. It was found that the spacing in all driving lanes followed the lognormal distribution checked by the K-S test. Figure 4 shows the statistical histograms and the probability density functions of the vehicle spacing. Table 4 shows the parameters of the fitted functions.

Depending on the freight volume of a truck, passing trucks are usually fully-loaded, empty-loaded, or overloaded. Therefore, statistical histograms of truck weights usually have multiple peaks. Herein, a Gaussian mixture distribution model (GMM) is utilized to fit the distribution of data with multiple peaks. The GMM uses a linear combination of several Gaussian probability distribution functions to fit the data distribution. The mathematical expression of the GMM is as follows:

(8) $$f(x|\theta )=\sum_{i=1}^{M}\omega_{i}\phi (x|\theta_{i})=\sum_{i=1}^{M}\omega_{i}\frac{1}{\sqrt{2\pi \sigma_{i}}}\exp\left(-\frac{{\left(x-\mu_{i}\right)}^{2}}{2\sigma_{i}^{2}}\right)$$

where $\;\omega_{i}$ is the weight coefficient of the *i*th Gaussian distribution, $0\leq \omega_{i}\leq 1$; the sum of all the weight coefficients is 1, that is $\;\sum_{i=1}^{M}\omega_{i}=1$, $\phi (x|\theta_{i})\;$is the density function of the *i*th Gaussian distribution; *x* is the vehicle weight or axle weight; $\mu_{i}$ is the mean of the *i*th normal distribution; and $\sigma_{i}^{2}$ is the variance of the *i*th normal distribution. Since $\;\omega_{i}$, $\sigma_{i}^{}$, and $\mu_{i}$ were unknown parameters in the model, the expectation maximization (EM) algorithm was used to solve the problem. This algorithm is a type of maximum likelihood estimation method. The likelihood function of the GMM model is written as follows:

(9) $$\mathrm{lg}\prod_{k=1}^{N}f(x|\theta )=\sum_{k=1}^{N}\mathrm{lg}f(x|\theta )$$

where the initial parameters are $\;\theta =(x_{i},\mu_{i},\sigma_{i})$, the specific steps to solve the problem are as follows:

1. Initialization of the parameter $\;\theta =(x_{i},\mu_{i},\sigma_{i})$—the K-means algorithm is used to cluster the sample data points, and the number of parameters and their corresponding values are selected according to the clustering results.
2. Step E—the proportion of each Gaussian function in the vehicle weight sample data is estimated. For a car weight sample value, the probability that it is combined by the *i*th Gaussian function is as follows:
  (10) $$\gamma (k,i)=\frac{x_{i}\cdot \phi (x|\mu_{i},\sigma_{i}^{2})}{\sum_{j=1}^{M}x_{j}\phi (x|\mu_{i},\sigma_{i}^{2})}$$
3. Step M—the parameter estimates corresponding to the likelihood function can be derived by the following:
  (11) $$\mu_{i}=\frac{1}{N_{i}}\sum_{k=1}^{N}\gamma (k,i)x_{k}$$
  (12) $$\sigma_{i}^{2}=\frac{1}{N_{i}}\sum_{k=1}^{N}\gamma (k,i)(x_{k}-\mu_{k})(x_{k}-\mu_{i})$$
  where $\;N_{i}=\sum_{k=1}^{N}\gamma (k,i)$, $\;\sum_{i=1}^{M}\omega_{i}=1$. The Lagrange multiplier is introduced into the GMM likelihood function, as follows: $\;\mathrm{lg}\prod_{k=1}^{N}f(x)+\lambda (\sum_{i=1}^{M}x_{i}-1)$. Once the function is found to reach the maximum value, the corresponding value is $\omega_{i}=N_{i}/N$.
4. The values of the likelihood function are calculated and checked for convergence. If it does not converge, iterations are performed for steps E and M. If it converges, the values corresponding to the parameters are the maximum likelihood estimates of each parameter.

It can be seen from the above formulae that the establishment of the GMM Gaussian mixed distribution model is a combination of multiple Gaussian distribution functions. The parameter that determines the number of Gaussian distribution functions is the number of Gaussian components, M. The selection of its optimal number is mostly determined by the Akachi information criterion (AIC), and the number of Gaussian components with the smallest AIC value is the optimal value [30,31]. The specific formula is written as follows:

(13) AIC=−2ln(L)+2M

(14) BIC=−2ln(L)+M×ln(n)

where *M* is the number of Gaussian components, *L* is the likelihood function, and *n* is the sample size.

**Figure 5:**
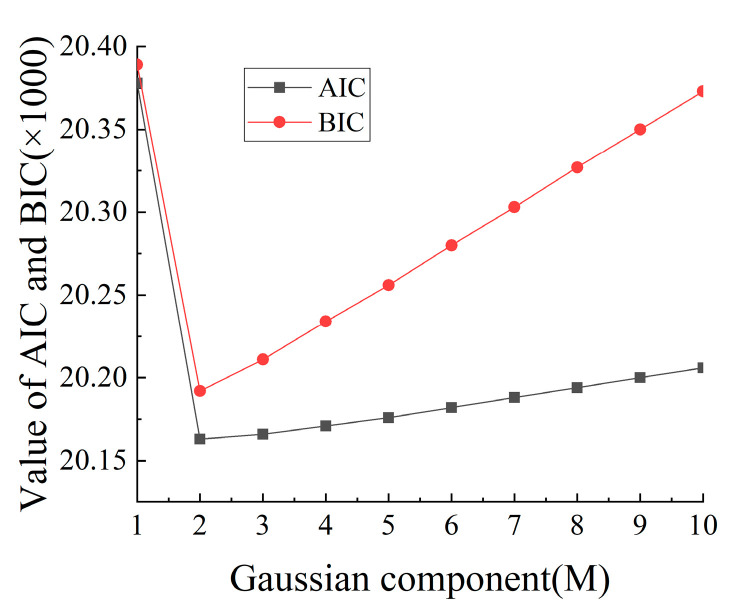
AIC and BIC curves of the GMM method, for C3 model’s weight.

Based on the vehicle weight data for July 2015, which was recorded by the WIM system of the Nanxi Yangtze River Bridge, the GMM method was used to simulate the total weight of each vehicle type. Taking the C3 model as an example, the AIC and BIC criteria were used to determine its optimal Gaussian component number. The results were calculated and shown in Figure 5.

As can be seen from Figure 5, the vehicle weight of the C3 model was optimal, based on the conditions of two-parameter Gaussian mixture model. The optimal number of Gaussian components and distribution parameters for the vehicle weights of the various vehicle types were calculated as shown in Table 5.

**Figure 6:**
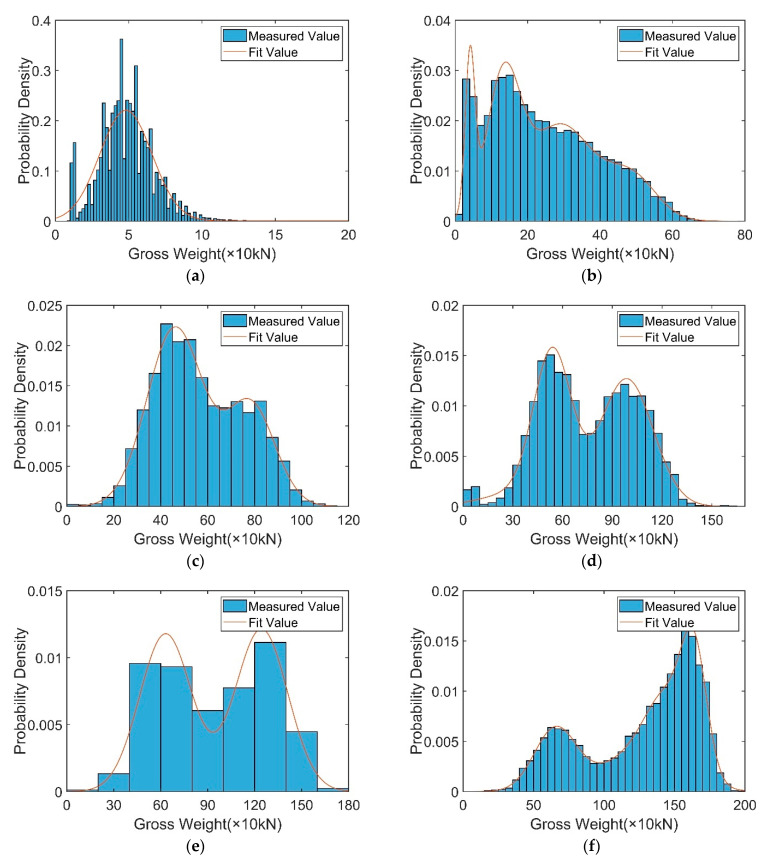
Probability distribution of vehicle weights for different vehicle types: (**a**) C1. (**b**) C2. (**c**) C3. (**d**) C4. (**e**) C5. (**f**) C6.

Table 4 shows the distribution parameter values for the Gaussian mixture distribution model, which can establish the probability density distribution functions of various vehicle types and can provide reliable sampling data for random traffic simulations. The probability distribution is shown in Figure 6.

**Figure 7:**
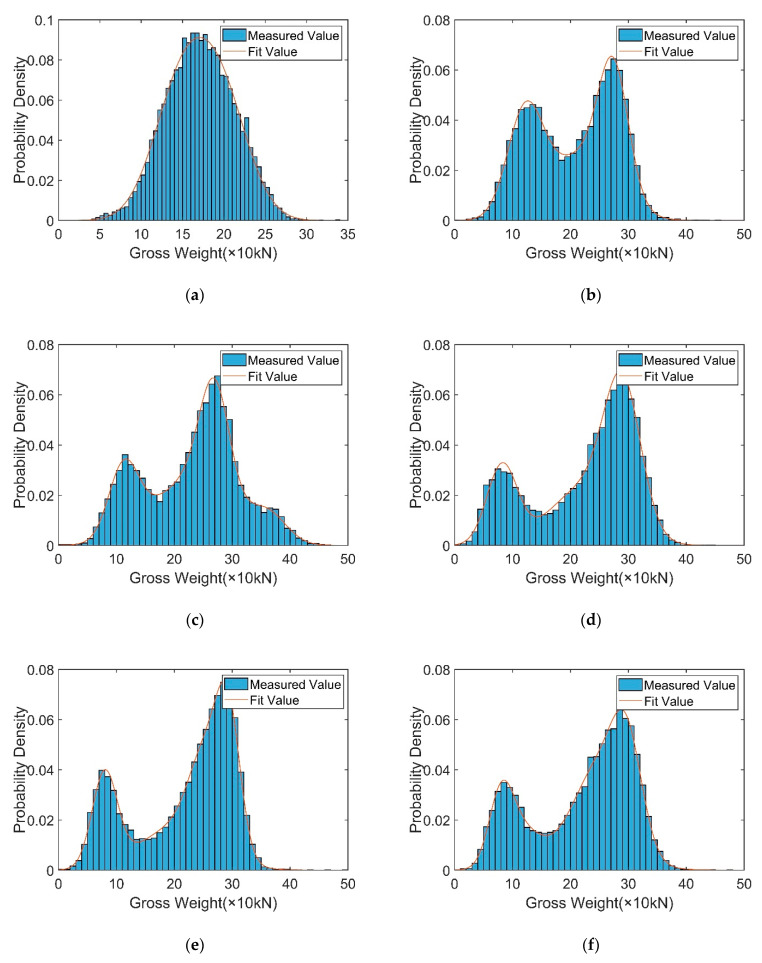
Probability distribution of C6 axle weights: (**a**) *AW*_61_. (**b**) *AW*_62_. (**c**) *AW*_63_. (**d**) *AW*_64_. (**e**) *AW*_65_. (**f**) *AW*_66_.

The probability distribution of each axle weight for every vehicle type was analyzed. Only the distribution parameters and the probability distribution diagrams of the axle weights of the six-axle vehicles are shown in Table 6 and Figure 7, where *AW_ij_* indicates the *j*th axis of the *i*th vehicle type.

### 3.3. Simulation of Vehicle Weight and Speed Considering Correlation

Based on the Copula function, the correlation between the vehicles’ weight and their driving speeds in the traffic flow parameters was analysed. Subsequently, stochastic traffic flow models that consider the parameter correlation were established. First, the three correlation coefficients: Pearson, Kendall, and Spearman are used to determine the correlation between the speed and the weight. The two-dimensional histogram of the vehicles’ weights and speeds was produced as follows:

1. Determine the marginal distribution of the vehicle speed and vehicle weight, and the specific data based on the Gaussian mixture model.
2. Evaluate the relevant parameters of the Copula function, based on the maximum likelihood estimation method.
3. Select an appropriate Copula function, according to the AIC.
4. Simulate the speed and weight samples for each vehicle type using the Monte Carlo (MC) method.

The relevant parameters and the AIC values of the five Copula functions for the vehicle speed and vehicle weight of C1 vehicles are shown in Table 7.

It can be observed from Table 7 that the t-Copula function had the smallest AIC value among the five Copula functions, followed by the Frank Copula function. For the other vehicle types, the Copula function and Copula-related parameter values that corresponded to the smallest AIC value were calculated separately. The results are shown in Table 8.

**Figure 8:**
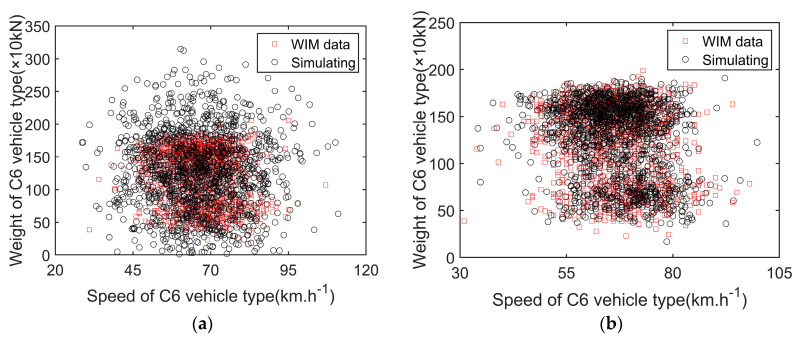
Comparison of measured data and simulated data of speed and weight of C6 type: (**a**) without correlation. (**b**)with correlation.

According to the expression of the estimated Copula function, combined with the use of the Monte Carlo method, two sets of data were simulated separately. The vehicle speed and weight data of the C6 type were taken as an example and compared with the measured data. Figure 8 shows the influence of parameter correlation on the samples. The simulated samples that considered correlation were more consistent with the measured data.

### 3.4. Stochastic Traffic Flow Load Modeling

**Figure 9:**
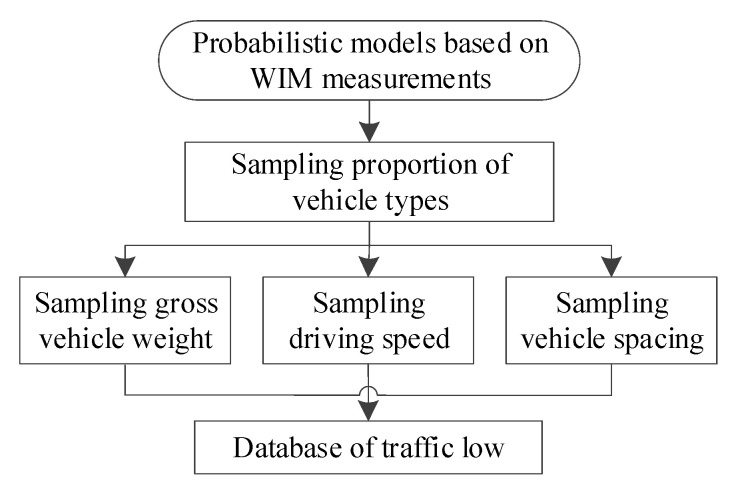
Flowchart for stochastic traffic flow simulation.

With the probability model of traffic parameters, the stochastic traffic flow can be simulated based on the Monte Carlo method. Subsequently, stochastic traffic flow samples can be formed by continuous sampling. The framework of the sampling method is summarized in Figure 9.

**Figure 10:**
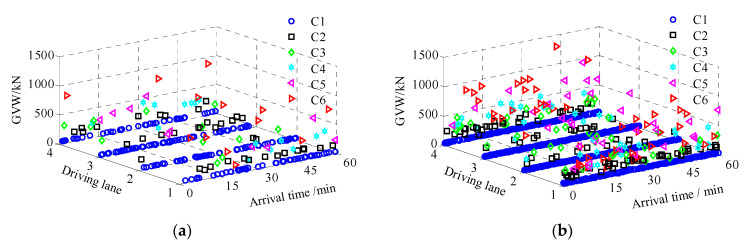
Simulated stochastic traffic loads: (**a**) present. (**b**) 20-years.

It should be noted that the traffic volume will increase with the growth of the local economy. The growth rate of traffic is normally considered to be different among different countries and cities. This study considered a relatively low annual growth rate of 2% for the current traffic. The simulated traffic flow over a duration of 60 min, both currently and in future projections, are shown in Figure 10.

In Figure 10, each symbol denotes a vehicle with a special mark. It was observed that there was a higher proportion of C1 vehicles. This was also the case in the measured traffic data. In addition, the maximum GVW almost reached 1500 kN, which is two times over the threshold for the legal vehicle weight.

## 4. Fatigue Stress Simulation of the Rib-to-Deck Welded Joints under Stochastic Traffic Loading

### 4.1. Engineering Prototype and Simulation

A typical steel bridge deck, on the steel box girder of the Nanxi Yangtze River Bridge, was taken as the engineering background. The numerical simulation of the fatigue crack propagation was conducted based on this bridge. The bridge deck parameters were as follows: the deck thickness was 16 mm; the U rib thickness was 8 mm; the width of the upper and lower parts of the rib were 300 mm and 170 mm, respectively; the transverse spacing of the U ribs was 600 mm; the diaphragm’s plate thickness was 600 mm; and the longitudinal spacing between two diaphragms was 3.2 m.

**Figure 11:**
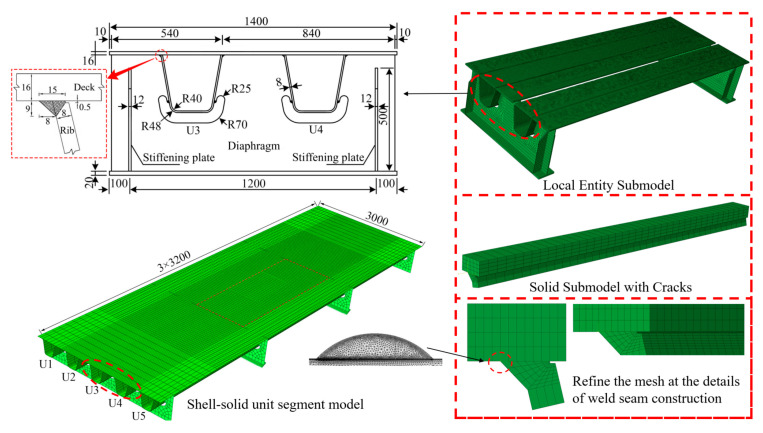
The finite element model of the prototype OSD.

To explore the fatigue stress state of the rib-to-deck welded joints of the bridge deck plates under the transverse distribution of the wheel tracks, the finite element model of the segment of the bridge deck plates was established, as shown in Figure 11. The model was composed of C3D8R elements and included two diaphragms. A steel Q345qD with an elastic modulus of 2.06 × 10^5^ MPa was selected, and the Poisson ratio was 0.3. The vertical displacement of the nodes on the diaphragm’s bottom plates was constrained to simulate the vertical constraint of the steel box girder on the segment model. Moreover, the horizontal displacement of the deck plate’s nodes in the transverse bridge direction was constrained to simulate the horizontal constraint of the steel box girder on the segment model. The longitudinal displacement of the nodes of the deck plates and the U ribs in the longitudinal bridge direction was also constrained to simulate the longitudinal constraint of the steel box girder on the segment model.

**Figure 12:**
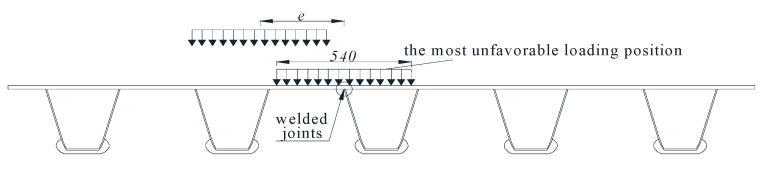
Lateral loading position of wheels (unit: mm).

Due to the longitudinal and transverse influences, the lines were extremely short; therefore, the simultaneous multiple truck effect had less impact on the fatigue damage. In other words, the fatigue damage that resulted from the simultaneous multiple truck load effect can be simplified as the superposition of a single truck load effect. A fatigue vehicle model with a double axels weight of 2 × 60 kN and a wheelbase of 1.2 m was selected for the subsequent computation. The superposition effect of the front and rear axis group was neglected. The landing area of one side wheel was 400 mm × 400 mm, and the landing area of another side wheel was 540 mm × 540 mm. As can be seen Figure 12, the adverse loading position was the transverse position, as this caused the maximum stress amplitude on the welded joints under the wheel load.

The moving step was defined by a 150 mm change in the lateral position of the wheel. The total number of steps considered in the model was seven. The moving loading of the wheels at the longitudinal bridge direction was simulated. The stress history curves of the fatigue of the welded joints under transverse distribution were obtained.

**Figure 13:**
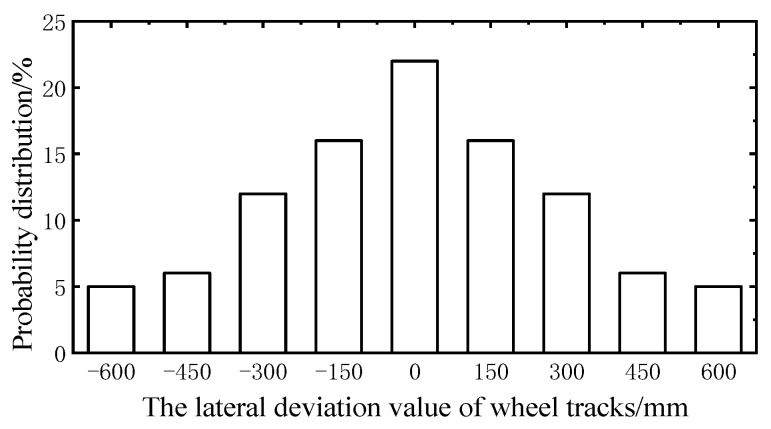
The transverse distribution frequency of wheel tracks.

According to the site-specific observation data, the wheel load basically acted within a transverse width of 1.2 m, and the lateral distribution of the wheel tracks in each lane was almost the same. This study selected the probability of the transverse distribution of domestic roads, which were obtained from the observation statistics of domestic researchers [32]. The transverse loading range of the wheel is 1.2 m. The lateral distribution was divided into nine parts, according to the 150 mm spacing. The distribution probability of each part is shown in Figure 13.

### 4.2. Influence of Transverse Vehicle Wheel Load on Structural Stress

**Figure 14:**
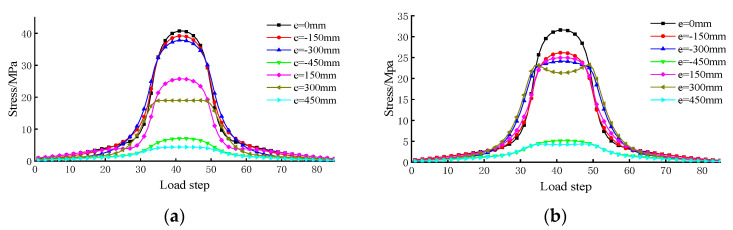
Stress history of rib-to-deck welded details under transverse distribution: (**a**) welded root. (**b**) welded toe.

The influence of the vehicle load in transverse distribution on the fatigue stress of rib-to-deck welded joints, with mid-span cross sections, was investigated. This study mainly focused on the stress influence lines of the welded root and toe. The numerical results are shown in Figure 14. It was observed that the stress was significantly affected by the transverse loading position. In addition, with the deviation distance between the center line of the tracks, the attention detail increased in the transverse bridge direction. Moreover, the shape of the stress influence line changed slightly, and the peak of the stress influence line decreased gradually.

To explore the influence of the transverse distribution of wheel tracks on the stress amplitude of rib-to-deck welded joints on steel bridge decks, this study used a transverse distribution model. In this model, the stress ranges of the welded joints, which were obtained from the different transverse loading positions of the wheels, are shown in Table 9. According to the Miner linear cumulative damage principle, the stress influence line and the distribution probability of the wheel tracks, which were obtained from the welded joints are the basic parameters of the equivalent stress amplitude under the transverse distribution of wheel tracks. The stress amplitude reduction coefficient is the ratio between the equivalent stress amplitude of the typical wheel track distribution model and the stress amplitude of the adverse loading model.

The equivalent stress range is written as follows:

(15) $$\Delta \sigma_{eq}={\left(\sum_{i=1}^{n}p_{i}\Delta \sigma_{i}^{m}\right)}^{1/m}$$

where *p_i_* is the distribution probability of the transverse deviation position of the *i*th wheel, $\Delta \sigma_{i}$ is the stress range for the transverse deviation position of the *i*th wheel, and *m* is the slope parameter of the S-N curve. The estimated stress reduction factors for the weld root and the toe were 0.8 and 0.78, respectively.

## 5. Random Propagation Characteristics of Fatigue Cracks at Rib-to-Deck Welded Joints under Realistic Traffic Loading

This section combines the stochastic traffic loading and the fatigue stress analysis method to conduct the stochastic propagation investigation. The adverse transverse loading position and the stress reduction factor was considered. The random propagation behaviors of fatigue cracks at the rib-to-deck weld root of steel bridge decks under stochastic traffic loading are also discussed here.

**Figure 15:**
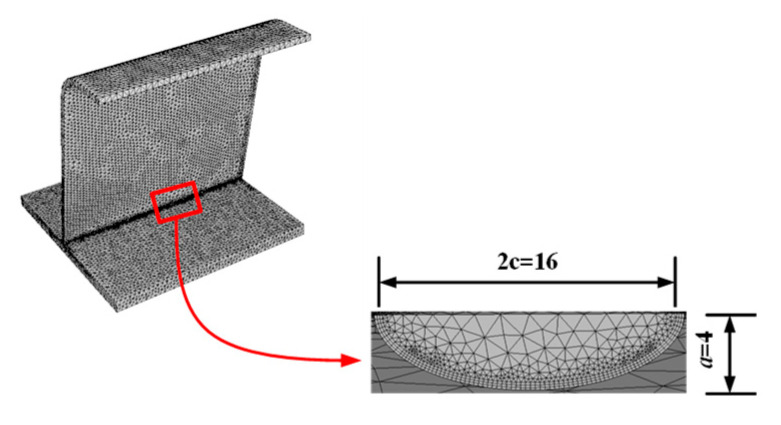
Re-meshed sub-model with a refined crack (unit: mm).

As mentioned, the crack depth, *a*, and the crack shape ratio, *a*/*c*, are two important indexes that affect the crack propagation behavior in the LEFM. The initial, semi-elliptic surface crack shape parameters were as follows: crack depth, *a* = 4 mm; surface length, 2*c* = 16 mm; and crack morphology ratio, *a*/*c* = 0.5. A crack-free sub-model of 300 mm × 300 mm × 280 mm, was cut from the midspan of the segment model. The crack and the sub-model were combined in FRANC3D, and the model was re-meshed with a scale characteristic of 0.4 mm. The re-meshed sub-model is shown in Figure 15.

**Figure 16:**
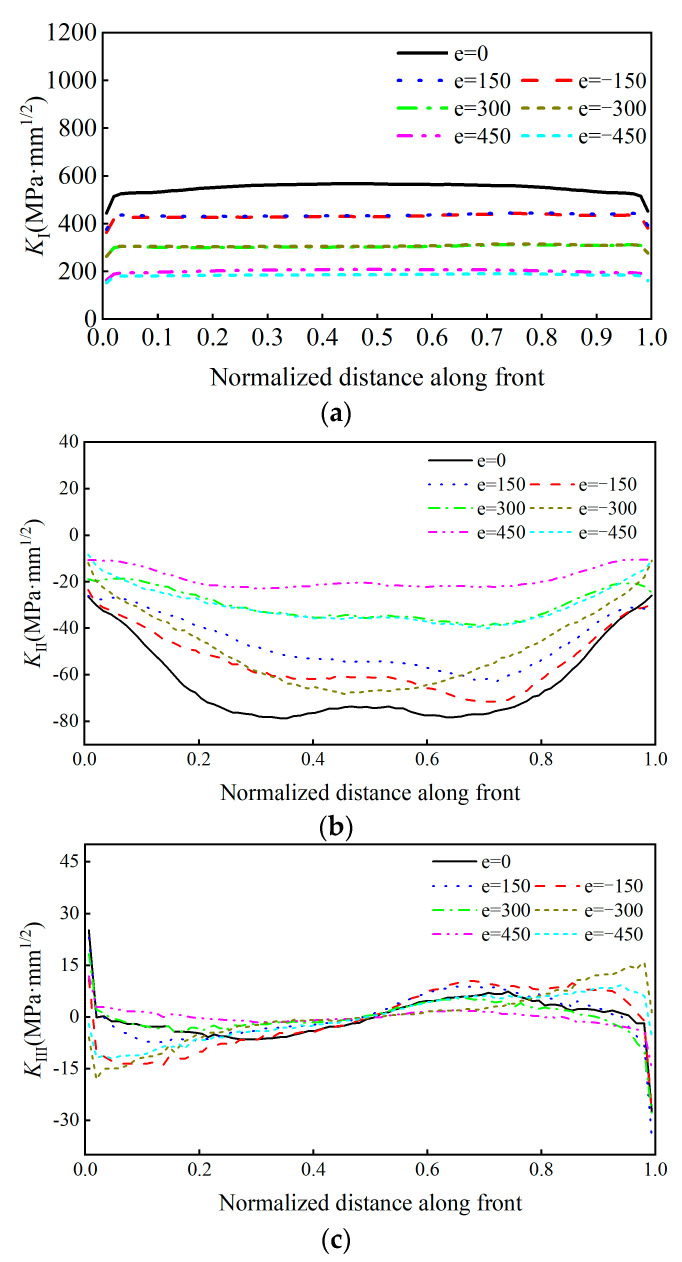
Stress intensity factor of crack tip at weld root of welded joints: (**a**) *K_I_*. (**b**) *K_II_*. (**c**) *K_III_*.

The analysis of the stress intensity of the crack tip at the rib-to-deck weld root, under an adverse loading position, longitudinally, is shown in Figure 16. The stress intensity factors of type-I, type-II, and type-III cracks are noted as *K_I_*, *K_II_*, and *K_III_*, respectively.

As can be observed from Figure 16, *K_I_* of the fatigue crack at the rib-to-deck welded root was significantly larger than *K_II_* and *K_III_*. Therefore, the composite crack was mostly affected by the type-I crack. Note that *K_I_* will decrease with the transverse moving from the adverse location. In addition, *K_II_* is negative to all transverse distributions, which restrains the sliding fatigue crack obviously. Considering the transverse distribution of wheel tracks, *K_II_* at the crack front was either positive or negative, and *K_I_* at the deepest crack point was insignificant.

**Figure 17:**
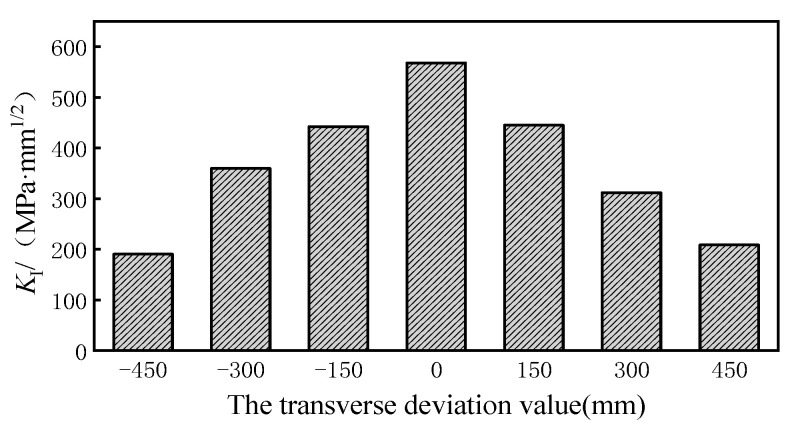
Comparison of maximum *K_I_* of fatigue crack at the weld root of the welded joints.

According to these results, type-I cracks cause fatigue cracks at the weld root. A comparative analysis was conducted to estimate the maximum stress intensity factor, *K_I_*, of the fatigue crack at the weld root under the transverse wheel loads. Figure 17 shows the stress intensity factor that was affected by the transverse loading position.

**Figure 18:**
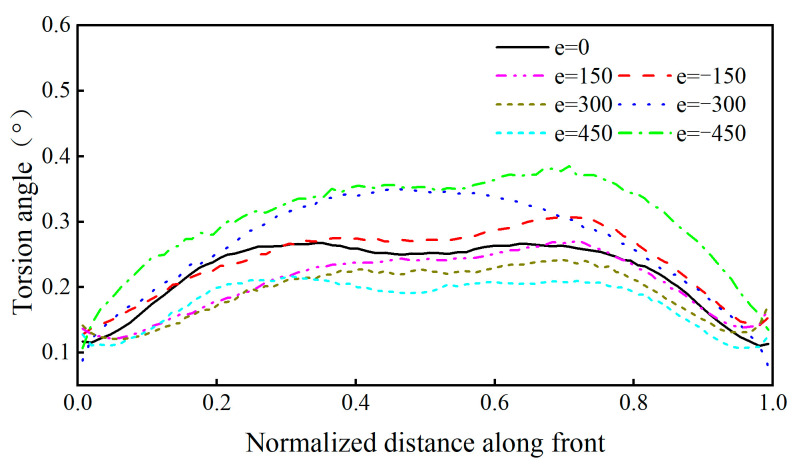
Propagation directions of the fatigue crack in weld root.

It can be observed from Figure 17 that the maximum value of *K_I_* was 568.18 (MPa·mm^1/2^) under the adverse transverse loading position. If the wheel tracks moved 450 mm to the right of the adverse loading position, the maximum *K_I_* decreases to 190.63 (MPa·mm^1/2^) with a reduction rate of 66.4%. The influence of the transverse distribution of the wheel tracks on the direction of the crack propagation is useful to understand the random crack propagation behavior of OSD cracks. The crack torsion angle at the weld root is shown in Figure 18 under the adverse loading condition.

It can be observed from Figure 18 that the propagation angle of the crack tip increased from 0.24° to 0.34°, which is an increase of 42%, under the condition of moving 450 mm transversally. Since the variation of the torsion angle in the middle part was negligible, the stochastic propagation behavior can be ignored. However, there were some differences in the propagation direction of the parts near the two ends. Under the effect of the transverse distribution of the wheel tracks, the torsional angle increased when the wheels moved to the right, but the torsional angle decreased when the wheels moved to the left.

**Figure 19:**
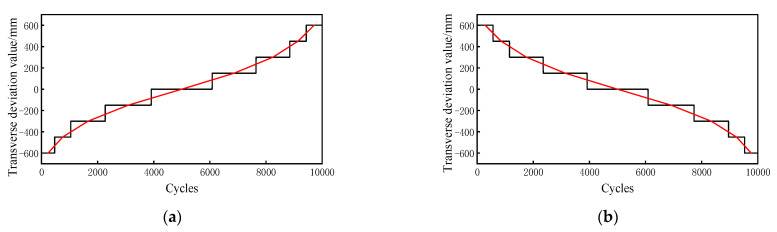
Load spectrum: (**a**) ascending load spectrum; (**b**) descending load spectrum.

Through the transformation of the wheels’ transverse loads, the transverse deviation value, *e*, of the wheel tracks was used as a random variable to analyze the random crack propagation path at the rib-to-deck weld root. The transverse distribution frequency is shown in Figure 19. MATLAB was used to simulate the transverse random wheel tracks to generate the stochastic traffic loading.

The random load spectra resulting from the stochastic traffic load was simplified in ascending and descending order to reflect the fatigue crack propagation behavior of the weld root of rib-to-deck welded joints more realistically and intuitively. The load spectra are shown in Figure 19. The random propagation paths under three load spectrum conditions were compared with that under the most adverse loading condition.

**Figure 20:**
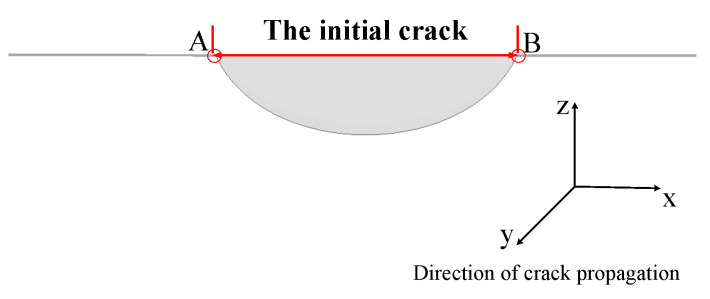
Location diagram of two endpoints, A and B, of the long axis of the crack.

**Figure 21:**
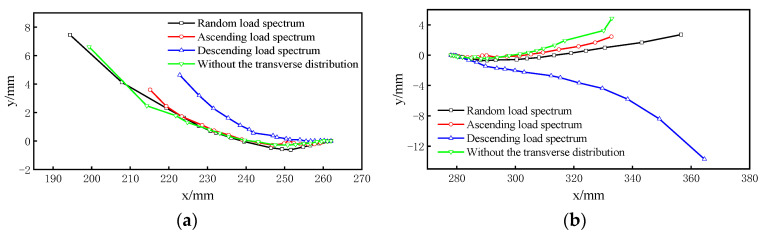
(**a**,**b**) The propagation paths of two endpoints, A and B, of the long axis of crack.

The propagation path of the fatigue crack was simulated based on the segment model established by FRANC3D-ABAQUS interaction technology. The basic parameters in the process of crack propagation were set. The parameters in Equation (1) are c = 5.21 × 10^−13^ and *n* = 3. The model was then submitted to ABAQUS for analysis. The propagation step, Δ*a*, of the midpoint of the front end of the fatigue crack was 0.6 mm. The propagation paths of the two endpoints, A and B, of the long axis of the semi-elliptic crack is discussed below. The location of the two endpoints is shown in Figure 20. The chosen number of propagation steps was 18 to clearly reflect the crack propagation paths and to ensure the efficiency and accuracy of the calculation. Figure 21 shows the propagation paths of the two endpoints.

In the random propagation paths at both ends of the semi-elliptic crack, the four paths had a small migration and almost coincided with each other within 10 mm of the propagation distance in the x axis direction. When the propagation distance in the x axis direction exceeded 10 mm, different degrees of migration began to appear, and the migration of the crack propagation path under the influence of a descending load spectrum, was more significant in the y axis. In addition, the propagation path under a random load spectrum was between the ascending load spectrum and the descending load spectrum.

## 6. Conclusions

A computational framework for the fatigue crack propagation of OSDs under stochastic traffic loads, based on the extended finite element model, have been presented in this study. The stochastic traffic load models were established based on site-specific weigh-in-motion measurements to simulate the fatigue stress spectra of welded joints. The influence of the transverse loading position of the wheel tracks on the stress intensity factor of the crack tip was also investigated. Furthermore, the random propagation paths of the crack under stochastic traffic loads were evaluated. Moreover, the influence of both the ascending and descending traffic loading patterns on the fatigue cracking behavior were also investigated. 

The numerical results indicated that the equivalent stress amplitudes of the weld root and weld toe of rib-to-deck welded joints under the random transversal wheel loading model were 0.80 and 0.78 times that of the traditional loading model, respectively. The maximum value of *K_I_* is 568.18 (MPa·mm1/2) under the most critical transversal condition of the wheel load. However, the maximum *K_I_* is 190.63 (MPa·mm1/2), a decrease of 66.4%, under the condition of transversal moving by 450 mm. The transverse distribution of the wheel tracks had a significant influence on the torsion angle of the crack. If the wheel was shifted 450 mm laterally from the most unfavorable position, the propagation angle of the crack tip would increase from 0.24° to 0.34° with an increase ratio of 42%. Under the three stochastic loads spectra and the transverse distribution of the wheel tracks, the propagation paths of the fatigue cracks almost coincided, where the value is within 10 mm of the propagation distance in the direction of the *x* axis. This phenomenon demonstrates that the migration occurred with different degrees. The migration effect is most significant under the descending load spectrum.

In addition to the fatigue cracks at the rib-to-deck weld root, the influence of the other typical fatigue factors of OSD needs to be further studied. The random fatigue crack propagation characteristics of steel bridge decks, under the combined action of weld defects and the transverse distribution of the wheel tracks, will be the focus of future research. In addition, the combination of the stochastic traffic model and the fatigue crack propagation behavior can be further developed.

## Figures and Tables

**Table 1:** Example of the traffic data recorded from the WIM system.

H: Min: S	Number	Type	Lane	Speed (km/h)	GVW (T)	Length (m)	Number of Axles	*Aw*_1_ (t)	*Aw*_2_ (t)	*Aw*_3_ (t)
00:02:27	1,651,570	K	2	79	0.9	3.9	2	0.5	0.3	
00:02:52	1,651,571	K	1	69	1.6	3.6	2	0.9	0.7	
00:03:41	1,651,572	A2	1	62	19.3	7.6	2	5.5	13.9	
00:04:51	1,651,573	A2	2	84	20.6	7.8	2	6.2	14.5	
00:04:52	1,651,574	H6	1	61	44.6	19.5	6	7.8	9.7	8.3
00:05:49	1,651,575	K	3	86	2.1	4.5	2	1.2	0.9	
00:08:12	1,651,576	K	1	117	3	4.9	2	1.6	1.4	
00:08:52	1,651,577	K	3	83	2	4.4	2	1.2	0.7	
00:09:16	1,651,578	C3	2	75	19.9	8.6	3	7.7	6.6	5.7

**Table 2:** Summary of the overloaded trucks.

Vehicle Type	C1	C2	C3	C4	C5	C6
Configurations	Light cars	Two-axle trucks	Three-axle trucks	Four-axle trucks	Five-axle trucks	Six-axle trucks
Average number of daily overloaded trucks	0	84	31	89	10	184
Average weight of overloaded trucks (t)	0	32	42	58	74	85

**Table 3:** Proportions of vehicle types in different driving lanes.

Vehicle Type	Lane 1 (%)	Lane 2 (%)	Lane 3 (%)	Lane 4 (%)
C1	79.58	94.01	96.64	59.83
C2	11.11	5.65	2.85	18.67
C3	1.10	0.02	0.09	2.36
C4	2.70	0.26	0.18	4.93
C5	0.22	0	0	0.38
C6	5.29	0.07	0.23	13.82

**Table 4:** Parameters of the fitted probability density functions.

Driving Lanes	Lane 1	Lane 2	Lane 3	Lane 4
Mean value *μ* (m)	5.4308	5.7520	5.5703	6.2548
Standard deviation *σ*	1.1701	1.3122	1.3384	1.2481

**Table 5:** Table of total refitting distribution parameters for each model.

Vehicle Type	Distribution Type	Distribution Parameter					
C1	2-GMM	$\omega_{1}$	0.02	$\mu_{1}$	17.90	$\sigma_{1}$	106.83
		$\omega_{2}$	0.98	$\mu_{2}$	4.81	$\sigma_{2}$	3.16
C2	5-GMM	$\omega_{1}$	0.27	$\mu_{1}$	34.51	$\sigma_{1}$	107.93
		$\omega_{2}$	0.29	$\mu_{2}$	12.40	$\sigma_{2}$	19.47
		$\omega_{3}$	0.11	$\mu_{3}$	48.83	$\sigma_{3}$	49.32
		$\omega_{4}$	0.23	$\mu_{4}$	24.10	$\sigma_{4}$	54.30
		$\omega_{5}$	0.10	$\mu_{5}$	3.88	$\sigma_{5}$	1.39
C3	2-GMM	$\omega_{1}$	0.33	$\mu_{1}$	78.19	$\sigma_{1}$	109.17
		$\omega_{2}$	0.67	$\mu_{2}$	46.11	$\sigma_{2}$	143.96
C4	3-GMM	$\omega_{1}$	0.34	$\mu_{1}$	65.97	$\sigma_{1}$	817.33
		$\omega_{2}$	0.30	$\mu_{2}$	53.07	$\sigma_{2}$	107.06
		$\omega_{3}$	0.36	$\mu_{3}$	101.76	$\sigma_{3}$	194.18
C5	2-GMM	$\omega_{1}$	0.51	$\mu_{1}$	124.16	$\sigma_{1}$	287.64
		$\omega_{2}$	0.49	$\mu_{2}$	62.87	$\sigma_{2}$	269.02
C6	4-GMM	$\omega_{1}$	0.23	$\mu_{1}$	144.92	$\sigma_{1}$	211.80
		$\omega_{2}$	0.23	$\mu_{2}$	65.59	$\sigma_{2}$	226.14
		$\omega_{3}$	0.27	$\mu_{3}$	127.01	$\sigma_{3}$	710.90
		$\omega_{4}$	0.27	$\mu_{4}$	163.92	$\sigma_{4}$	83.23

**Table 6:** Fitting parameter of each axle load of the six-axle vehicles.

Vehicle Type	GMM Type	Parameters					
*AW*_61_	2-GMM	$\omega_{1}$	0.56	$\mu_{1}$	14.93	*σ*1	11.64
		$\omega_{2}$	0.44	$\mu_{2}$	19.85	*σ*2	10.57
*AW*_62_	3-GMM	$\omega_{1}$	0.32	$\mu_{1}$	27.45	$\sigma_{1}$	6.68
		$\omega_{2}$	0.32	$\mu_{2}$	12.21	*σ*2	9.61
		$\omega_{3}$	0.36	$\mu_{3}$	21.91	*σ*3	31.43
*AW*_63_	5-GMM	$\omega_{1}$	0.31	$\mu_{1}$	27.13	$\sigma_{1}$	7.27
		$\omega_{2}$	0.19	$\mu_{2}$	11.33	*σ*2	6.88
		$\omega_{3}$	0.22	$\mu_{3}$	24.02	*σ*3	25.57
		$\omega_{4}$	0.11	$\mu_{4}$	36.10	*σ*4	10.50
		$\omega_{5}$	0.17	$\mu_{5}$	19.13	*σ*5	36.89
*AW*_64_	3-GMM	$\omega_{1}$	0.45	$\mu_{1}$	28.94	$\sigma_{1}$	10.20
		$\omega_{2}$	0.21	$\mu_{2}$	8.19	*σ*2	6.87
		$\omega_{3}$	0.34	$\mu_{3}$	22.24	*σ*3	37.25
*AW*_65_	4-GMM	$\omega_{1}$	0.20	$\mu_{1}$	7.97	$\sigma_{1}$	4.65
		$\omega_{2}$	0.28	$\mu_{2}$	29.30	*σ*2	4.46
		$\omega_{3}$	0.26	$\mu_{3}$	19.88	*σ*3	43.00
		$\omega_{4}$	0.26	$\mu_{4}$	25.39	*σ*4	9.13
*AW*_66_	5-GMM	$\omega_{1}$	0.28	$\mu_{1}$	26.02	$\sigma_{1}$	11.70
		$\omega_{2}$	0.20	$\mu_{2}$	8.68	*σ*2	5.83
		$\omega_{3}$	0.14	$\mu_{3}$	18.75	*σ*3	28.64
		$\omega_{4}$	0.14	$\mu_{4}$	22.74	*σ*4	51.20
		$\omega_{5}$	0.24	$\mu_{5}$	30.17	*σ*5	6.45

**Table 7:** Parameters of driving speeds and gross weights of C1 vehicles.

Vehicle Type	Parameter Value	Gaussian	t	Gumbel	Frank	Clayton
C1	α	0.0527	0.0593	1.0334	0.3704	0.0672
	AIC	−548.8	−1573.3	−647.3	−728.8	−687.1

**Table 8:** Minimum AIC values and copula parameter values for each vehicle type.

Parameter Value	C2(2)	C3	C4	C5	C6
α	−0.0728	−0.9543	−0.1502	−0.1349	−1.0241
AIC	−430.2	−53.9	−136.8	−15.9	−338.6
Copula	t	Frank	t	t	Frank

**Table 9:** Stress amplitude at rib-to-deck welded joints of steel bridge deck under transverse distribution of wheel tracks.

Transverse Position (mm)	Stress Range (MPa)		Transverse Distribution Proportion
	Weld Root	Weld Toe	
0	40.77	31.59	22%
−150	39.20	26.15	16%
−300	37.85	24.12	12%
−450	7.09	5.14	6%
150	25.75	24.97	16%
300	19.01	21.35	12%
450	4.39	4.20	6%

Note: in the transverse position, 0 represents the adverse loading position, a negative value represents the wheel track’s deviation to the right and a positive value represents the wheel track’s deviation to the left.

## Data Availability

The data presented in this study are available on request from the corresponding author.
